# Interdisciplinary Collaboration and Job Satisfaction Among Doctors and Nurses in Greek Public Hospitals: A Cross-Sectional Study

**DOI:** 10.7759/cureus.88712

**Published:** 2025-07-24

**Authors:** Konstantinos Flindris, Stefanos Flindris, Freideriki Nteka, Athanasios Kaliardas

**Affiliations:** 1 Ophthalmology, General Hospital of Ioannina "G. Hatzikosta", Ioannina, GRC; 2 2nd Department of Obstetrics and Gynecology, General Hospital of Thessaloniki "Ippokrateio", Thessaloniki, GRC; 3 Cardiac Surgery, University General Hospital of Ioannina, Ioannina, GRC

**Keywords:** doctor-nurse collaboration, greek public hospitals, interdisciplinary collaboration, interprofessional communication, level of job satisfaction, public health policy

## Abstract

Background/purpose: Effective collaboration between doctors and nurses is linked to better staff morale and patient outcomes. However, nurses often perceive teamwork as poorer than doctors do. In Greek public hospitals, resource constraints make such collaboration critical, yet data are limited. This study examined doctor-nurse collaboration and job satisfaction in Greece and how they relate.

Methods: We conducted a cross-sectional survey of 113 doctors and 188 nurses (n = 301) in six Greek public hospitals. Participants completed an anonymous questionnaire including a physician-nurse collaboration scale (CCPNQ) and a job satisfaction scale (KUHJSS), along with demographics and years of experience. Group differences were analyzed with t-tests, and correlations and multiple linear regression were used to assess associations between collaboration, satisfaction, and tenure.

Results: Nurses rated collaboration quality significantly lower than doctors (mean score 3.66 ± 0.55 vs. 3.85 ± 0.54, p = 0.004), but reported higher job satisfaction (3.78 ± 0.72 vs. 3.45 ± 0.64, p = 0.007). Collaboration scores were moderately correlated with job satisfaction for both nurses (r = +0.558, p < 0.001) and doctors (r = +0.495, p < 0.001). Among nurses, years of service correlated negatively with satisfaction (r = -0.498, p < 0.001), indicating lower satisfaction in more experienced nurses; no such trend appeared for doctors. In regression analysis, collaboration remained a strong independent predictor of higher job satisfaction (p < 0.001) when controlling for profession and experience, while longer tenure predicted lower satisfaction primarily among nurses.

Conclusion: Strong interdisciplinary collaboration was closely linked to higher job satisfaction for both nurses and doctors. However, nurses’ poorer perceptions of collaboration and the drop in satisfaction among veteran nurses highlight areas for improvement. Interventions to enhance doctor-nurse communication, teamwork, and shared decision-making could improve job satisfaction and help retain experienced staff. Fostering a collaborative culture in Greek hospitals may benefit healthcare providers' well-being and, ultimately, patient care.

## Introduction

High-quality patient care depends not only on individual expertise but also on effective teamwork between healthcare professionals [1]. In particular, collaboration between physicians and nurses - often termed interdisciplinary or interprofessional collaboration - is crucial for safe, efficient healthcare delivery [2]. Extensive research highlights that strong doctor-nurse collaboration can improve workplace efficiency, enhance staff job satisfaction, and even lead to better patient outcomes [3]. Conversely, poor communication and teamwork across these roles can undermine care quality and contribute to professional frustration or burnout [4].

Despite broad acknowledgment of its importance, achieving optimal doctor-nurse collaboration remains a challenge. Historically, this relationship has been hierarchical, with doctors in dominant decision-making roles and nurses in subordinate roles [5]. Many nurses found this traditional model restrictive and demeaning, leading to workplace dissatisfaction. While modern healthcare has evolved toward more team-based approaches, remnants of this culture persist and hinder interdisciplinary collaboration [6].

Notably, prior studies have found that nurses and physicians often perceive their collaboration very differently. For instance, doctors tend to rate the quality of collaboration with nurses higher than nurses [7]. Such discrepancies suggest that nurses may feel less included or valued in teamwork processes compared to their colleagues. These perception gaps are important, as they could influence job morale and indicate underlying communication issues [8].

Job satisfaction of healthcare providers is itself a critical outcome for health systems. Satisfied staff are more productive, provide higher quality care, and are less likely to leave their jobs, mitigating turnover and staffing shortages. In nursing, especially, low job satisfaction has been linked to burnout and intent to quit, exacerbating the global nursing shortage [9]. It stands to reason that when nurses feel respected and effectively collaborate in patient care, their work experience improves. Similarly, for doctors, working in a collegial team environment may enhance their own job fulfillment and reduce stress [10].

While the relationship between interdisciplinary collaboration and job satisfaction has been examined in various international contexts, there is limited data from the Greek healthcare setting. Greece’s public hospitals operate in a context of constrained resources and high demands, where effective teamwork is essential [11]. Understanding how Greek doctors and nurses view collaboration with each other, and how this relates to their job satisfaction, can help identify needs for organizational improvement [12].

This study aimed to investigate interdisciplinary collaboration and job satisfaction among doctors and nurses in six Greek public hospitals and the associated factors. By addressing these questions, the study aims to provide evidence to guide interventions for improving teamwork and supporting healthcare professionals in Greek hospital settings, leading to superior patient outcomes.

## Materials and methods

A cross-sectional survey was conducted across six Greek public hospitals (General Hospital of Athens “Elpis”, General Hospital of Thessaloniki “Papageorgiou”, General Hospital of Ioannina “G. Hatzikosta”, University General Hospital of Ioannina, General Hospital of Arta, and General Hospital of Kerkira). The hospitals were selected to represent a variety of settings (including large urban tertiary hospitals and smaller regional hospitals) in order to improve the generalizability of findings. Data collection took place over a three-month period (from November 2024 to January 2025). During this time, doctors and nurses in various departments of the selected hospitals were invited to participate in the study by completing an anonymous questionnaire. Participation was voluntary, and informed consent was obtained from all respondents. The study protocol was reviewed and approved by the appropriate institutional ethics committees of each hospital (protocol ID: 15656, date of approval: 11/11/2024), and all procedures conformed to the principles of the Declaration of Helsinki.

Licensed doctors and registered nurses of any specialty or department within the hospitals were included in the survey. Both staff with direct patient-care roles and those in supervisory roles were eligible, as long as they had regular interactions with the other profession (doctor or nurse) in their work. Investigators distributed questionnaires during staff meetings, shifts, or via the hospitals’ internal communication channels. To encourage a high response rate, reminders were given, and completion was allowed during work breaks. No personal identifiers were collected, ensuring confidentiality. The respondent group included a mix of junior and senior staff. Based on self-reports, the participants’ years of professional experience ranged widely (from less than one year to over 30 years in practice). Perceived collaboration between doctors and nurses was measured independently using the Communication and Collaboration among Physicians and Nurses Questionnaire (CCPNQ) (see Appendix A). This instrument, originally developed by Vazirani et al. (2005) [13], assesses the quality of doctor-nurse interactions and teamwork in the clinical setting. It contains items evaluating aspects such as communication effectiveness, mutual respect, problem-solving, and teamwork efficiency as experienced between the two professional groups. Participants rate statements on a Likert-type scale (e.g., from “strongly disagree” to “strongly agree” or frequency ratings), with higher scores indicating a perception of better collaboration. The instrument has been used in prior studies of interdisciplinary collaboration and has shown good psychometric properties. For our study, we used the Greek version of the CCPNQ, which has been formally translated and validated in the Greek language and healthcare settings. Job satisfaction was evaluated using the Kuopio University Hospital Job Satisfaction Scale (KUHJSS) (see Appendix B). The KUHJSS was originally developed in Finland and covers sundry dimensions of healthcare employees’ job satisfaction (such as leadership, working environment, motivational factors, and team spirit) [14]. We utilized the Greek version of the KUHJSS, which has also been formally translated and validated in Greek healthcare settings. The scale asks respondents to indicate their level of satisfaction with different facets of their job on a Likert scale, typically ranging from “very dissatisfied” to “very satisfied.” An overall job satisfaction score can be derived by summing or averaging item scores, with higher scores representing greater overall satisfaction. The questionnaire packet also collected demographic and professional information. Key variables included the respondent’s profession (doctor or nurse), hospital (site of employment), years of professional experience in healthcare, age, gender, highest educational qualification, and salary range. The internal consistency of the two main scales (collaboration and job satisfaction) was assessed in our sample by Cronbach’s alpha.

Statistical analysis was implemented using IBM SPSS Statistics for Windows, Version 28.0 (released 2021, IBM Corp., Armonk, NY). We used descriptive statistics to summarize the sample characteristics and key variables. Mean and standard deviation (SD) were calculated for continuous variables, and frequencies and percentages for categorical variables. An independent samples t-test was used to compare the mean collaboration score reported by doctors versus that reported by nurses. Given that the collaboration questionnaire form was slightly different for each group, we ensured comparability by using equivalent items and scoring ranges; a higher score always denoted better perceived collaboration. We also compared mean job satisfaction scores between doctors and nurses using a t-test. Before using t-tests, we checked the normality of score distributions with the Shapiro-Wilk test and by inspecting histograms. The collaboration and satisfaction scores were approximately normally distributed; thus, t-tests were deemed appropriate. In cases where distributions were skewed or variances unequal, the non-parametric Mann-Whitney U test was alternatively applied. Correlations were assessed to evaluate our primary hypotheses about associations. Pearson’s correlation coefficient (r) was used to measure the linear relationship between the collaboration score and the job satisfaction score in an overall and subgroup (profession, hospital site) analysis. Similarly, we examined the correlation between sundry-associated factors, e.g., years of service and job satisfaction. If variables were not normally distributed or if there were outliers, Spearman’s rank correlation (ρ) was additionally checked for consistency.

Multiple linear regression analyses were conducted to further explore predictive relationships while controlling for potential confounders. In the primary regression model, we treated the overall job satisfaction score as the dependent variable. As key independent variables, we included the interdisciplinary collaboration score and years of service. We also included a dummy variable for profession (0 = nurse, 1 = physician) to adjust for any baseline differences between doctors and nurses, and an interaction term between collaboration and profession was tested to see if the association between collaboration and satisfaction differed by profession. The regression assumptions (linear relationship, homoscedasticity, normality of residuals, multicollinearity) were checked and satisfied; variance inflation factors (VIFs) for the predictors were all below 2, indicating no serious multicollinearity. We ran this regression on the combined sample. In addition, we performed separate subgroup regressions for nurses and for physicians to identify any differences in predictors within each group. In those subgroup models, we entered collaboration score and years of service as predictors of job satisfaction. For completeness, we also examined whether demographic factors like age or hospital site needed to be controlled for; if initial analyses suggested any significant effect of hospital or age on satisfaction, we would include them, but in our data, these did not markedly alter the results, so the final models were parsimonious.

A subgroup analysis by hospital was carried out to ensure that pooling data across the six hospitals was justifiable. We compared the mean collaboration and job satisfaction scores across hospitals (using one-way ANOVA, with Tukey post-hoc tests) to see if any hospital had outlier values. We also repeated the correlation analysis within each hospital’s subset of respondents to observe if the collaboration-satisfaction relationship held consistently. While sample sizes per hospital (~50 on average) limited statistical power to detect subtle differences, this analysis was useful for checking the robustness of the overall trends.

For all statistical tests, a two-tailed p-value < 0.05 was considered the threshold for statistical significance. We report exact p-values where relevant and provide 95% confidence intervals (CIs) for key estimates to indicate the precision of our findings.

## Results

A total of 301 healthcare professionals completed the survey (response rate 86%, since 350 questionnaires were distributed). Of the respondents, 113 (37.5%) were doctors and 188 (62.5%) were nurses. The doctors included both consultants/attendings and resident doctors from departments such as Internal Medicine, Surgery, Pediatrics, Radiology, Ophthalmology, and Intensive Care Unit (ICU), while the nurse group included staff nurses from medical, surgical, ICU, and outpatient units, as well as a few nurse managers. The mean age of doctors was approximately 34.64 ± 9.78 years, while nurses were slightly older than doctors (42.64 ± 12.10 years), reflecting that many physicians in the sample were resident doctors. The majority of nurses were female (n = 152, 80.9%), whereas the physician group had a slight male majority (n = 65, 57,5%). This gender distribution is typical in Greek hospitals. The majority of participants were occupied in University General Hospital of Ioannina (n = 70, 23.3%), followed by General Hospital of Arta (n = 57, 18.9%), General Hospital of Athens “Elpis” (n = 53, 17.6%), General Hospital of Kerkira (n = 43, 14.3%), General Hospital of Ioannina “G. Hatzikosta” (n = 40, 13.3%), and General Hospital of Thessaloniki “Papageorgiou” (n = 38, 12.6%).

Doctors in our sample had substantially more clinical tenure than nurses. On average, doctors reported 15 ± 9 years of experience, compared with 10 ± 9 years for nurses (p < 0.001). The vast majority of doctors had up to 10 years of work experience in healthcare (n = 79, 69.9%), while almost one out of two nurses had over 20 years of service in healthcare (n = 91, 48.2%), consistent with their age differences and career stages (Table 1).

**Table 1:** Descriptive statistics of the participants

Variable	Doctors (n = 113)	Nurses (n = 188)	Summary (n = 301)
Profession (%)	113 (62.5%)	188 (37.5%)	301 (100%)
Female (%)	48 (42.5%)	152 (80.9%)	200 (66.4%)
Age (mean ± SD)	34.64 ± 9.78	42.64 ± 12.1	39.6 ± 11.9
Years of service (mean ± SD)	15 ± 9	10 ± 9	12 ± 9

No significant differences were observed in the key outcome variables (collaboration perception and job satisfaction) by gender or by hospital site in preliminary analyses. While there was some variability between hospitals, for instance, General Hospital of Athens “Elpis,” had a slightly higher overall job satisfaction mean than the other Greek Public Hospitals; these differences were not statistically significant in ANOVA (p = 0.15 for satisfaction differences across the six hospitals, p = 0.22 for collaboration score differences across hospitals). Therefore, data were combined for subsequent analyses, with hospital site considered as a random effect that did not require further adjustment.

**Figure 1:**
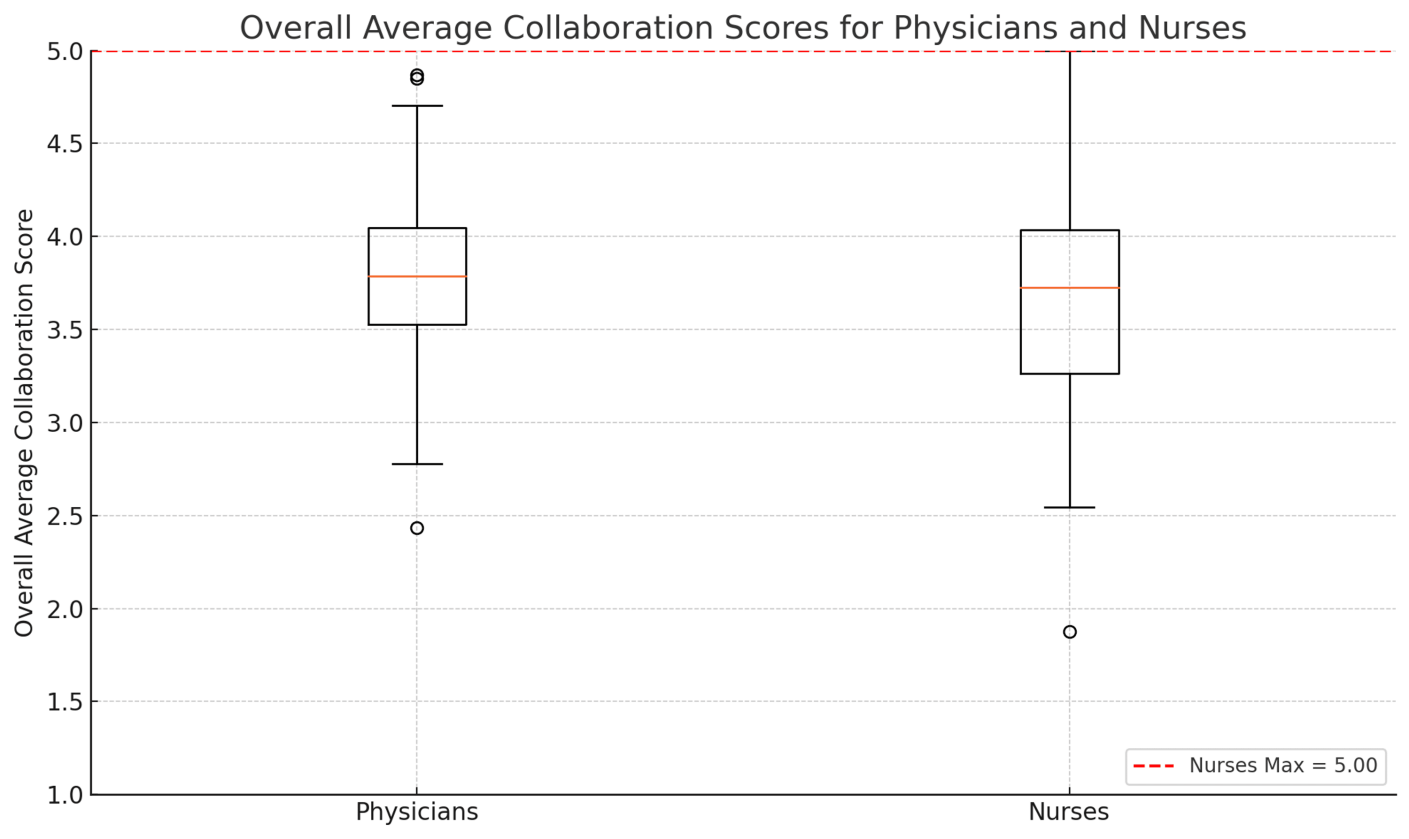
Boxplot of overall average collaboration scores for physicians and nurses, displaying the median (center line), interquartile range (box), and 1.5 × IQR whiskers of each group’s average score on the Communication and Collaboration among Physicians and Nurses questionnaire (scale range 1–5). The red dashed line marks the maximum observed score for nurses (5). The legend reports the result of an independent-samples Welch’s t-test (t = 2.90, df ≈ 239, p = 0.004), indicating a statistically significant difference between doctors and nurses.

First, we examined how doctors and nurses rated the quality of collaboration in their work. In our study, CCPNQ showed high reliability, with Cronbach’s α values of 0.888 for doctors and 0.921 for nurses for all scale versions, indicating high internal consistency. These values are in line with previously reported reliability for the Greek version of CCPNQ. There was a clear disparity between the two groups’ perceptions. Doctors generally rated physician-nurse collaboration quite positively, whereas nurses gave less favorable ratings. On the CCPNQ, doctors reported a mean collaboration score of 3.85 ± 0.54 on a 1-5 scale, with an interquartile range (IQR) of 0.5 (with 5 indicating excellent collaboration), compared to nurses’ mean of 3.66 ± 0.55, with an IQR of 0.8. This difference was statistically significant (independent t-test, t (df = 239) = 2.9, p = 0.004) (Figure 1). In practical terms, this suggests that doctors often feel the communication and teamwork with nurses is of high quality, whereas nurses perceive more often communication gaps or teamwork deficiencies.

In our study, the reliability of the KUHJSS in our data was excellent, with Cronbach’s α = 0.903 for doctors and 0.944 for nurses, affirming that the scale provided a coherent measure of satisfaction. These values are in line with previously reported reliability for the Greek version of the KUHJSS. Overall job satisfaction levels among the participants were moderate to high. On the KUHJSS, the mean job satisfaction score in the entire sample was 3.66 ± 0.71, suggesting that, on average, the respondents were “somewhat satisfied” to “satisfied” with their jobs. However, there was considerable individual variability: about 5% of the participants had scores indicating low satisfaction (e.g., <3 on the 1-5 scale), while roughly 53% reported very high satisfaction (>4 on the scale).

**Figure 2:**
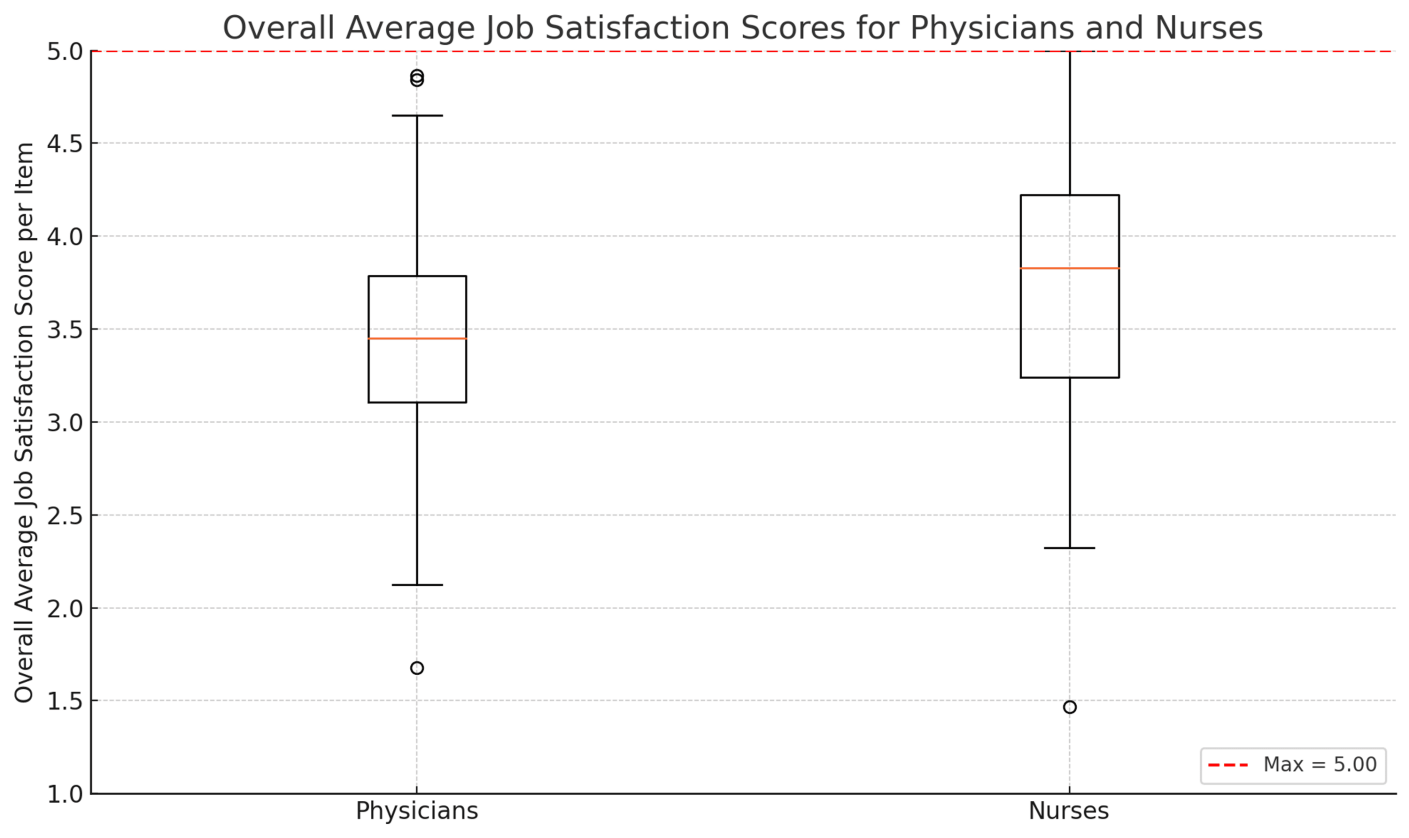
Boxplot of overall average job satisfaction scores for physicians and nurses, presenting the median (center line), interquartile range (box), and 1.5 × IQR whiskers of each group’s average score on the KUHJSS questionnaire (1–5 scale). The red dashed line marks the maximum observed per-item average for nurses (5). The legend reports the Welch’s t-test result (t = 2.75, df ≈ 287, p = 0.007), indicating a statistically significant difference between doctors and nurses. KUHJSS: Kuopio University Hospital Job Satisfaction Scale, IQR: interquartile range

Comparing the two professions, doctors and nurses presented a statistically significant difference in overall job satisfaction mean scores (t (df = 287) = 2.75, p = 0.007). Physicians’ mean satisfaction score was 3.45 ± 0.64, with an IQR of 0.6, and nurses’ mean was 3.78 ± 0.72, with an IQR of 0.8 (Figure 2). These results indicate that nurses report greater job satisfaction than physicians in our sample.

Our results strongly indicate that better perceived collaboration is associated with higher job satisfaction. In the total sample of 301 participants, the Pearson correlation coefficient between the collaboration score and the job satisfaction score was r = +0.42 (p < 0.001). This represents a moderate positive correlation: generally, those who reported stronger physician-nurse collaboration tended to also report greater satisfaction in their work.

**Figure 3:**
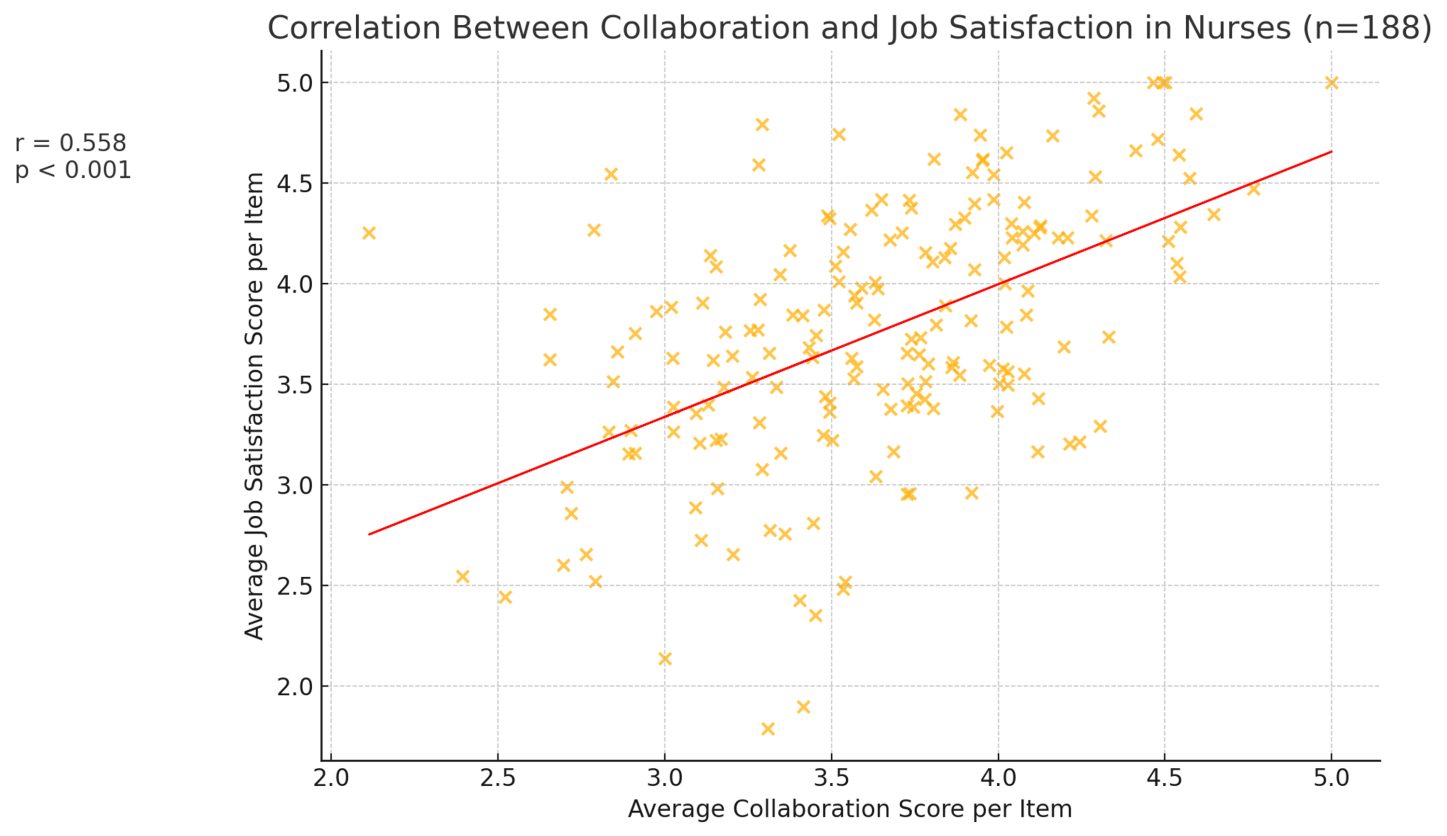
Scatter plot, showing the correlation between average collaboration and job satisfaction scores per item for nurses (n = 188). The red line is the best-fit regression line, and the annotation shows r = 0.558, p < 0.001, reflecting the strong positive association.

**Figure 4:**
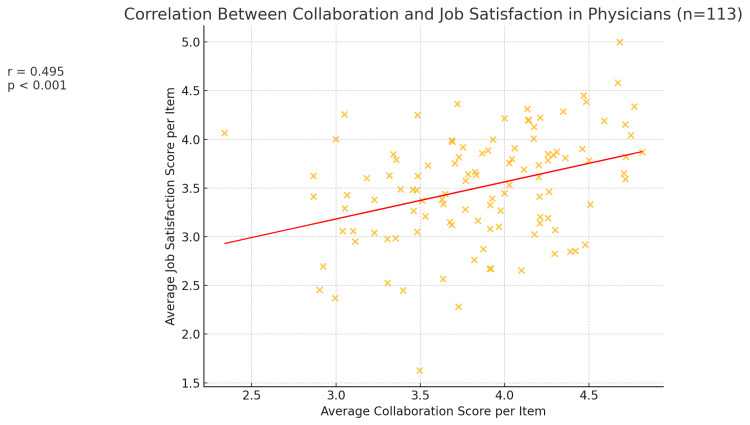
Scatter plot, showing the correlation between the average collaboration and job satisfaction scores per item for doctors (n = 113). The red line is the best-fit regression line, and the annotation shows r = 0.495, p < 0.001, reflecting the positive association.

When stratified by professional group, this positive association persisted in both subgroups. Among nurses (n = 188), the correlation between collaboration and job satisfaction was r = +0.558 (p < 0.001) (Figure 3). Nurses who felt they had good communication and teamwork with physicians were much more likely to be satisfied with their jobs. In contrast, nurses who perceived poor collaboration tended to be dissatisfied or only marginally satisfied in their roles. Similarly, for physicians (n = 113), the correlation was r = +0.495 (p < 0.001) (Figure 4), indicating a significant positive relationship, albeit slightly weaker than that for nurses. This suggests that while the link between collaboration and satisfaction is evident for both groups, it may be particularly pronounced for nurses. One possible interpretation is that collaboration quality might constitute a larger portion of what makes nurses satisfied at work, whereas physicians’ job satisfaction, though still influenced by teamwork, might also strongly depend on other factors, like autonomy or administrative load.

**Figure 5:**
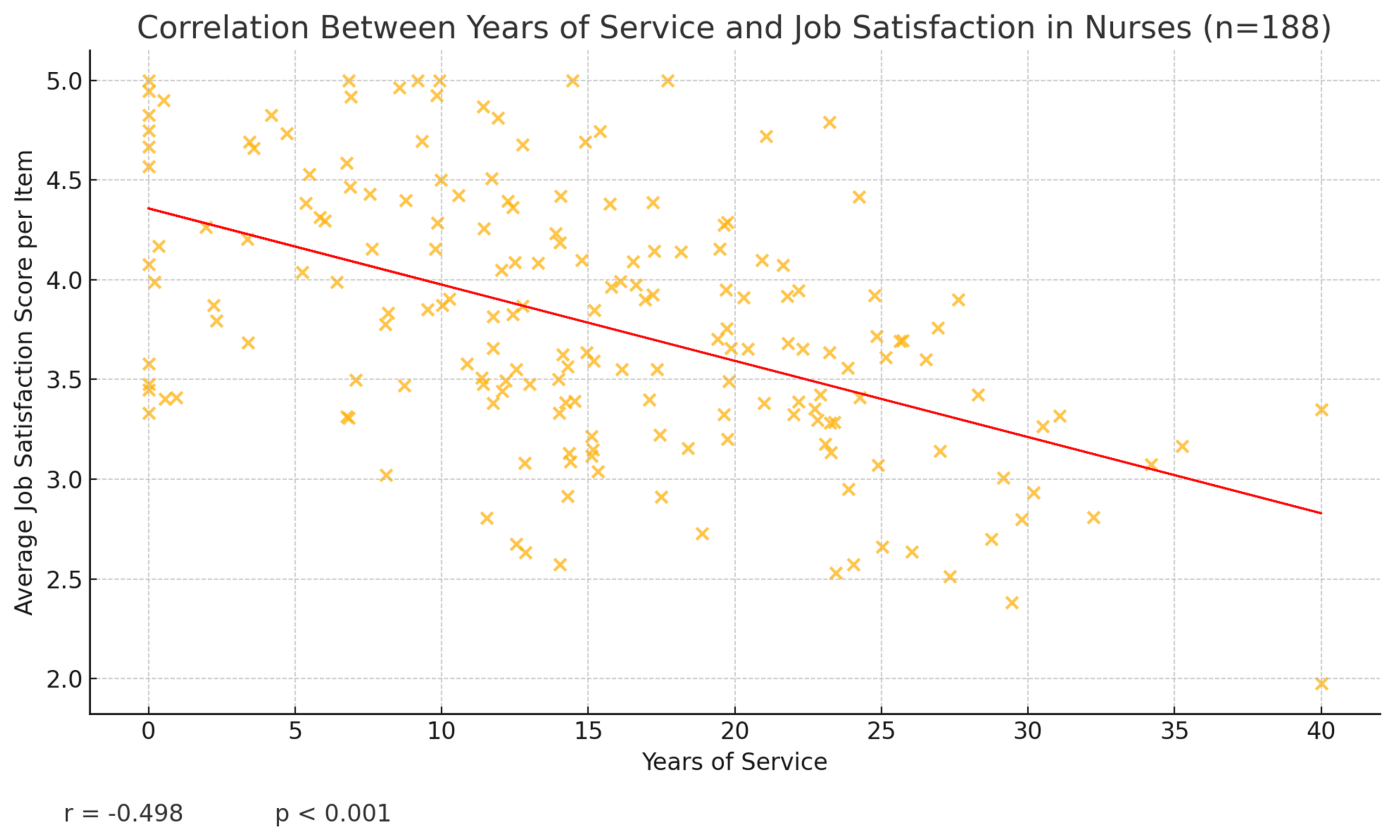
Scatter plot showing the correlation between years of service and average job satisfaction score per item for nurses (n = 188). The red line is the best-fit regression line, and the annotation displays r = –0.498, p < 0.001, indicating a significant negative relationship.

Another finding of interest emerged regarding work experience. We found that among nurses, greater years of service were associated with lower job satisfaction. The Pearson correlation between years of professional experience and the job satisfaction score was r = -0.498 (p < 0.001) (Figure 5), indicating a modest but significant negative correlation. In practical terms, nurses who had been working longer tended to report lower satisfaction levels than those who were relatively early in their careers.

By contrast, for physicians, there was no statistically significant correlation between years of service and job satisfaction (r = -0.097, p = 0.27). It should be noted that we did not find significant differences in collaboration perceptions by years of service, i.e., older vs. younger nurses rated collaboration with doctors similarly on average. Hence, the decline in satisfaction for nurses with tenure is not simply due to a change in how they view collaboration; it likely involves other evolving job factors.

Multiple linear regression analyses were performed to determine the independent contributions of collaboration and experience to job satisfaction. In the combined sample model (including both doctors and nurses, n = 301), we regressed job satisfaction score on collaboration score, years of service, profession (doctor vs. nurse), and an interaction term (Collaboration × Profession). This model was significant (F-test p < 0.0001) and explained about 25% of the variance in job satisfaction (adjusted R² ≈ 0.25), indicating a moderately good fit for an attitudinal study.

Collaboration emerged as a strong independent predictor of job satisfaction (standardized β = +0.433, p < 0.001). Profession (nurse vs. doctor) by itself was not a significant predictor when collaboration and other factors were in the model (p = 0.18), implying that any baseline difference between doctors’ and nurses’ satisfaction was explained by the other variables. The interaction term between collaboration and profession was not significant (p = 0.40), suggesting that the effect of collaboration on satisfaction did not significantly differ in strength between doctors and nurses.

Years of service had an independent negative effect on job satisfaction (β = -0.15, p = 0.03) in the combined model. This indicates that, controlling for collaboration and profession, greater experience still slightly predicted lower satisfaction overall. However, given our earlier observation of this mainly in nurses, separate regressions for each group were performed.

In the nurse-only regression, collaboration score and years of service together explained around 20% of the variance in nurses’ job satisfaction (adjusted R² = 0.20, p < 0.001 for the model). For nurses, both predictors were significant: collaboration had a positive β = +0.489 (p < 0.001), and years of service had a β = -0.226 (p = 0.005). This means that even when considering both factors at once, a nurse’s perception of good collaboration with doctors is a major contributor to being satisfied, and having many years on the job has an independent, mild association with being less satisfied. No other variables (like age or education level) added significant explanatory power for nurses.

For physicians, the physician-only regression with collaboration and years yielded an adjusted R² of about 0.10 (10% variance explained, p = 0.01 for the model). In that model, collaboration was a significant predictor (β = +0.304, p = 0.004), whereas years of service was not significant (β = -0.101, p = 0.30), reinforcing that for doctors, perceived teamwork with nurses does relate to satisfaction, but tenure doesn’t play a notable role in their satisfaction level.

These analyses affirm the earlier findings: perceived interdisciplinary collaboration is a key factor linked to job satisfaction in both professions, and long tenure has a detrimental association with satisfaction primarily for nurses.

## Discussion

In this cross-sectional study of doctors and nurses in six Greek public hospitals, we found clear evidence that interdisciplinary collaboration and job satisfaction are positively interrelated. Healthcare professionals who reported better communication and teamwork between doctors and nurses also tended to be more satisfied with their jobs. Our findings echo those of prior studies internationally, strengthening the generalization that when nurses and doctors collaborate effectively, it boosts morale [15]. Our study extends this knowledge to the Greek public hospital context, suggesting that the benefits of interdisciplinary collaboration on job satisfaction are not limited by country or healthcare system differences.

Importantly, we observed that nurses and doctors do not perceive collaboration equally. This divergence is not surprising and aligns with numerous reports in the literature. Studies across various settings have documented that doctors often overestimate the quality of teamwork compared to nurses’ assessments [16]. Our data provide a quantitative confirmation of this phenomenon in ward and hospital settings in Greece. This difference in perspective stems from the traditional hierarchy in healthcare teams: doctors, who typically lead decision-making, feel that interactions are smooth because they are in a position of authority, whereas nurses feel their input is not adequately sought or valued, leading to a less positive view of “collaboration.” While modern practice is improving, elements of that hierarchy likely persist, contributing to our finding that nurses still perceive collaboration as suboptimal relative to doctors’ perceptions [17]. This perceptual gap highlights that hospital administrators and team leaders should not assume that a lack of complaints from doctors means all is well - they should actively solicit and listen to nurses’ perspectives on teamwork. Our results suggest that nurses desire stronger collaboration than they currently experience [18]. Given that job satisfaction is tied to turnover intentions, fostering a collaborative environment might help mitigate staff turnover, especially among nurses who are often at higher risk of burnout [19].

Another key finding was the negative relationship between years of service and job satisfaction in nurses. This suggests that more experienced nurses in Greek hospitals tend to be less satisfied with their jobs. There are several possible reasons for this trend. One explanation could be burnout accumulation - over years of working in a high-stress, under-resourced environment, nurses may become fatigued, leading to lower satisfaction [20]. Another factor might be career stagnation; in many healthcare systems, including Greece’s, nurses may have limited opportunities for advancement or role change over decades, leading to frustration or a feeling of being undervalued. Additionally, organizational changes and austerity measures in the Greek public sector over the last decade might have disproportionately affected veteran nurses (e.g., wage freezes, increased workloads), thereby eroding their job satisfaction [21]. Interestingly, literature on the impact of age/experience on nurse satisfaction is mixed. Some studies find older nurses are more content, possibly because those who remain are the ones who have adapted or genuinely love their work, whereas others find younger nurses are more satisfied, aligning with our result [22]. Our findings lean toward the latter - they raise concern that if the current younger nurses do not see improvements in their work conditions and interdisciplinary relationships. This has implications for workforce planning: hospitals should implement strategies to maintain and even boost satisfaction as nurses gain experience. Examples could include offering professional development opportunities (specialist training, promotions to advanced practice or leadership roles), recognizing and rewarding long-serving nurses, and ensuring adequate staffing to reduce burnout for tenured staff [23].

By contrast, doctors’ job satisfaction did not significantly decline with years of service in our study. This difference between professions could be due to a variety of factors. Doctors often have clearer career trajectories and may attain senior positions that give them more autonomy, authority, and possibly better financial rewards over time [24]. These factors might sustain or even increase satisfaction for some, offsetting the toll of years of work stress. In addition, by the time doctors reach later career stages, those who were extremely dissatisfied may have left clinical practice (or moved to the private sector), whereas many nurses might feel economically or otherwise bound to remain despite dissatisfaction. Our findings suggest that interventions to improve satisfaction may need to be tailored: for nurses, targeting the mid-career slump with support and new challenges might be key, whereas for doctors, ensuring early-career support and adequate work-life balance might be more relevant.

Our findings suggest that improving collaboration could be a highly effective lever for enhancing job satisfaction across the board. Potential initiatives to improve interdisciplinary collaboration include team training programs, where doctors and nurses learn communication and teamwork skills together. Another approach is to implement joint decision-making forums [25]. Hospitals might also review their policies and culture: encouraging a more egalitarian culture where appropriate, addressing instances of disrespect or poor communication swiftly, and perhaps establishing mentorship pairings across professions to foster mutual understanding. By closing the collaboration perception gap and truly improving teamwork, not only should satisfaction rise, but patient care is likely to benefit from the more cohesive team dynamic. Prior research has indeed linked better nurse-physician collaboration to improved patient outcomes and safety, such as lower error rates and higher patient satisfaction [25]. Thus, our findings contribute to a larger narrative: collaborative practice is a win-win for staff and patients.

We must also consider the context of Greek public hospitals. These hospitals have faced significant challenges in recent years, including economic constraints, staffing shortages, and high patient loads. Under such conditions, both collaboration and job satisfaction might be strained. The fact that in our study, average job satisfaction was moderate suggests there is room for improvement. Greek health authorities and hospital administrators should heed signs of discontent, especially among nursing staff. Retention of experienced nurses is critical - if dissatisfaction drives many to early retirement or migration to the private sector/abroad, the healthcare system loses valuable expertise. Addressing issues that matter to staff, such as ensuring respectful interdisciplinary relationships, could improve retention [26]. In addition, investing in organizational structures that facilitate teamwork, like regular interdisciplinary meetings, adequate nurse-to-doctor ratios, and collaborative leadership models, may strengthen the overall work environment [27].

We acknowledge several limitations in this study. First, the cross-sectional design prevents us from drawing conclusions about causality. While it is tempting to interpret that better collaboration leads to higher satisfaction, it is also plausible that more satisfied individuals perceive their work environment more positively, or that a third factor (like effective leadership in a unit) independently boosts both collaboration and satisfaction. Longitudinal studies or intervention trials would be needed to untangle cause and effect. Second, our data are based on self-reported questionnaires, which can introduce response biases. Participants might have answered in socially desirable ways, although the anonymity and the fact that we did see a range of positive and negative responses suggest that most answered honestly. There is also the potential for common method bias since both key variables (collaboration and satisfaction) were reported by the same respondents in the same survey; this could artificially inflate the correlation between them. We attempted to mitigate this by assuring respondents that there were no right or wrong answers and by separating the questionnaires in the survey packet. Another limitation is representativeness. We sampled six public hospitals, but these may not capture all types of hospital environments in Greece. Thus, generalization should be cautious - the culture and satisfaction levels in private or rural healthcare settings might differ. Moreover, within each hospital, we used convenience sampling. It is possible that those who chose to respond have systematically different. Our decent sample size and similarity of results across hospitals are reassuring, but sampling bias cannot be fully excluded. We also had a higher proportion of nurses than doctors participating. This partly reflects the workforce composition, but it could also be that nurses were more interested in voicing their opinions on this topic, whereas some doctors might have been less engaged in the survey. If some segment of doctors hardly responded, the physician data might tilt towards those who had time/interest, perhaps affecting average responses. However, our physician group did include a wide mix of specialties and both junior and senior doctors, lending some confidence that we captured a reasonable cross-section of physician perspectives. In addition, while we controlled for profession and years of service, there could be other confounding variables we didn’t measure that influence job satisfaction. For example, personality traits or personal life stressors can affect how someone feels about their job. Departmental differences might also play a role; we did not stratify deeply by department due to sample size limits, but ICU nurses, for instance, might have different collaboration dynamics with physicians compared to ward nurses. Future research with larger samples could explore such nuances.

Despite limitations, our study has notable strengths. It is, to our knowledge, one of the first multi-center studies in Greece to quantitatively assess nurse-doctor collaboration alongside job satisfaction for both groups. The inclusion of both nurses and physicians in the same study allows direct comparison of perceptions and a more holistic picture of the interdisciplinary climate. We used well-validated instruments, including a job satisfaction scale specifically validated in Greek, which adds credibility to our measures. The consistency of our core findings with theoretical expectations and prior studies’ results lends support to the validity of our conclusions. Furthermore, by spanning six hospitals, we improved variability in organizational culture and reduced the chance that results are due to one idiosyncratic site, yet we found remarkably consistent patterns across sites. This consistency strengthens the argument that these are fundamental relationships at play, not mere local anomalies.

## Conclusions

In summary, this study demonstrates a significant positive link between interdisciplinary collaboration and job satisfaction among doctors and nurses in Greek public hospitals. Healthcare professionals who perceive better communication and teamwork between nurses and physicians tend to be much more satisfied with their jobs, an observation that holds true for both nurses and doctors. Conversely, nurses in particular report lower satisfaction when collaboration is poor, and worryingly, nurses with longer years of service appear to have diminished job satisfaction. We also found that nurses consistently perceive the quality of doctor-nurse collaboration to be lower than physicians perceive it, highlighting a gap in experiences and expectations that needs to be addressed.

These findings carry important implications. Improving the nurse-physician collaborative dynamic is a key strategy for enhancing staff morale, reducing burnout, and retaining experienced healthcare workers. Hospital administrators and clinical leaders in Greece and elsewhere should recognize that investing in interdisciplinary collaboration (through training, culture change, and supportive policies) can yield dividends in staff satisfaction and stability. In particular, efforts to give nurses a stronger voice in clinical teamwork and to educate physicians in teamwork and communication could help bridge the perception gap. Likewise, special attention should be given to supporting seasoned nurses, who in our study were less satisfied - interventions such as advanced career pathways, mentorship roles, and burnout prevention programs could help maintain their engagement and satisfaction.

Ultimately, a hospital environment where doctors and nurses collaborate as true partners is likely to not only make the workplace more gratifying for those professionals but also to improve patient care. Satisfied, well-coordinated teams are better positioned to provide high-quality, safe care to patients. Our study adds evidence from the Greek healthcare setting that aligns with this principle. We encourage healthcare institutions to regularly assess their teamwork climate and job satisfaction levels (perhaps through surveys similar to those used in this study) as a diagnostic tool. Addressing any identified weaknesses in interdisciplinary collaboration should be seen as a pathway to strengthening the health system’s human resource foundation. Future research could build on these findings by exploring interventions to enhance collaboration and by examining the impact of improved collaboration on objective outcomes such as staff turnover rates and patient satisfaction in the Greek context. In the meantime, the message is clear: fostering a collaborative culture between nurses and doctors is not only beneficial for patient outcomes but is also integral to the job satisfaction and sustainability of the healthcare workforce.

## Appendices

Appendix A 

**Table 2:** Communication and Collaboration among Physicians and Nurses Questionnaire

Interdisciplinary collaboration among physicians and nurses This section is answered by doctors and nurses.						
		Strongly disagree	Disagree	Neither agree nor disagree	Agree	Strongly agree
1	The doctor shows the nurse respect in the presence of the patients' parents and companions.	1	2	3	4	5
2	The doctor is informed by the nurse about the patient's condition.	1	2	3	4	5
3	The doctor-nurse relationship ensures collaboration.	1	2	3	4	5
4	The doctor trusts the nurse's work.	1	2	3	4	5
5	The doctor accepts the nurse's decisions regarding the patient's care.	1	2	3	4	5
6	The nurse feels an important part of the patient's care team.	1	2	3	4	5
7	The doctor fairly evaluates the nurse's work.	1	2	3	4	5
8	The doctor collaborates with the nurse for therapeutic treatment and decision management.	1	2	3	4	5
9	The doctor shows sensitivity to the nurse's family situation.	1	2	3	4	5
10	The doctor shows sensitivity to the nurse's personal needs.	1	2	3	4	5
11	The physician accepts the nurse's responsibility in patient care.	1	2	3	4	5
12	The doctor assesses the nurse's administrative skills.	1	2	3	4	5
13	The doctor accepts the nurse's opinion regarding treatment and management decisions.	1	2	3	4	5
This section is answered only by nurses						
14	Sometimes I am not properly informed by the doctors in the department.	1	2	3	4	5
15	Communication with doctors is very good.	1	2	3	4	5
16	Sometimes it is necessary to check the accuracy of the order given to me.	1	2	3	4	5
17	I am interested in communicating with the doctors of the department.	1	2	3	4	5
18	It is easy to ask for instructions from the department's doctor on duty.	1	2	3	4	5
19	The time I have been working in the department has been a great experience for me.	1	2	3	4	5
20	This department applies a multidimensional approach to patient care.	1	2	3	4	5
21	Nurses participate in decision-making.	1	2	3	4	5
22	There is collaboration between doctors and nurses in decision-making.	1	2	3	4	5
23	There is communication between doctors and nurses before decisions are made.	1	2	3	4	5
24	There is open communication between doctors and nurses when making decisions.	1	2	3	4	5
25	I was informed about the patient's condition when needed.	1	2	3	4	5
26	I was informed promptly when the patient's condition changed.	1	2	3	4	5
27	I noticed an unjustified delay in informing me about the patient's condition.	1	2	3	4	5
28	Nurses call doctors on a regular basis for issues related to patient care	1	2	3	4	5

Appendix B 

**Table 3:** Kuopio University Hospital Job Satisfaction Scale

This section is answered by doctors and nurses.						
		Strongly disagree	Disagree	Neither agree nor disagree	Agree	Strongly agree
1	My supervisor is genuinely interested in the well-being of staff.	1	2	3	4	5
2	My supervisor treats staff fairly and equally.	1	2	3	4	5
3	My supervisor provides staff with feedback to develop their work.	1	2	3	4	5
4	My supervisor keeps me well informed about issues related to my unit.	1	2	3	4	5
5	My supervisor provides opportunities for continuous professional development for staff.	1	2	3	4	5
6	My supervisor encourages staff to take part in planning the operation of our unit.	1	2	3	4	5
7	My supervisor is interested in work performance and results.	1	2	3	4	5
8	Working in my department is safe.	1	2	3	4	5
9	Working in my department is comfortable.	1	2	3	4	5
10	The workload is evenly distributed across my department.	1	2	3	4	5
11	My workload is appropriate.	1	2	3	4	5
12	I have the ability to plan my work independently.	1	2	3	4	5
13	I have the ability to make independent decisions in my work.	1	2	3	4	5
14	The hospital's senior management appreciates my work.	1	2	3	4	5
15	My department has the appropriate equipment to ensure quality care.	1	2	3	4	5
16	My salary is appropriate in relation to the demands of my job.	1	2	3	4	5
17	My department has the appropriate work facilities.	1	2	3	4	5
18	My job duties are challenging.	1	2	3	4	5
19	I can apply a wide range of my skills/experiences to my work.	1	2	3	4	5
20	The patient’s response motivates me in my work.	1	2	3	4	5
21	My work is interesting.	1	2	3	4	5
22	I am willing to work in the hospital setting in the future.	1	2	3	4	5
23	I value my work.	1	2	3	4	5
24	The combination of work and personal life is successful.	1	2	3	4	5
25	I trust the experience of colleagues.	1	2	3	4	5
26	There is good team spirit in my department.	1	2	3	4	5
27	The flow of information works well in my department.	1	2	3	4	5

## References

[1] Chamberlain-Salaun J, Mills J, Usher K (2013). Terminology used to describe health care teams: an integrative review of the literature. J Multidiscip Healthc.

[2] Almost J, Doran DM, McGillis Hall L, Spence Laschinger HK (2010). Antecedents and consequences of intra-group conflict among nurses. J Nurs Manag.

[3] Bruzzese JM, Usseglio J, Goldberg J, Begg MD, Larson EL (2020). Professional development outcomes associated with interdisciplinary research: an integrative review. Nurs Outlook.

[4] Reader TW, Flin R, Mearns K, Cuthbertson BH (2007). Interdisciplinary communication in the intensive care unit. Br J Anaesth.

[5] Lancaster G, Kolakowsky-Hayner S, Kovacich J, Greer-Williams N (2015). Interdisciplinary communication and collaboration among physicians, nurses, and unlicensed assistive personnel. J Nurs Scholarsh.

[6] Karam M, Brault I, Van Durme T, Macq J (2018). Comparing interprofessional and interorganizational collaboration in healthcare: A systematic review of the qualitative research. Int J Nurs Stud.

[7] Zwarenstein M, Goldman J, Reeves S (2009). Interprofessional collaboration: effects of practice-based interventions on professional practice and healthcare outcomes. Cochrane Database Syst Rev.

[8] Chang WY, Ma JC, Chiu HT, Lin KC, Lee PH (2009). Job satisfaction and perceptions of quality of patient care, collaboration and teamwork in acute care hospitals. J Adv Nurs.

[9] Tomajan K (2012). Advocating for nurses and nursing. Online J Issues Nurs.

[10] Lodh P, Ghosh S (2022). Doctors’ work life quality and effect on job satisfaction: an exploratory study based on literature review. Pakistan J Multidiscip Innov.

[11] Keith AC, Warshawsky N, Neff D, Loerzel V, Parchment J (2021). Factors that influence nurse manager job satisfaction: an integrated literature review. J Nurs Manag.

[12] Lee SE, Dahinten SV, MacPhee M (2016). Psychometric evaluation of the McCloskey/Mueller Satisfaction Scale. Jpn J Nurs Sci.

[13] Vazirani S, Hays RD, Shapiro MF, Cowan M (2005). Effect of a multidisciplinary intervention on communication and collaboration among physicians and nurses. Am J Crit Care.

[14] Kvist T, Mäntynen R, Partanen P, Turunen H, Miettinen M, Vehviläinen-Julkunen K (2012). The job satisfaction of finnish nursing staff: the development of a job satisfaction scale and survey results. Nurs Res Pract.

[15] Busari JO, Moll FM, Duits AJ (2017). Understanding the impact of interprofessional collaboration on the quality of care: a case report from a small-scale resource limited health care environment. J Multidiscip Healthc.

[16] Dahlke S, Hunter KF, Reshef Kalogirou M, Negrin K, Fox M, Wagg A (2020). Perspectives about interprofessional collaboration and patient-centred care. Can J Aging.

[17] Melo MB, Barbosa MA, Souza PR (2011). Job satisfaction of nursing staff: integrative review. Rev Lat Am Enfermagem.

[18] Dinius J, Philipp R, Ernstmann N (2020). Inter-professional teamwork and its association with patient safety in German hospitals-a cross sectional study. PLoS One.

[19] Kabbash IA, El-Sallamy RM, Abdo SA, Atalla AO (2020). Job satisfaction among physicians in secondary and tertiary medical care levels. Environ Sci Pollut Res Int.

[20] Youngwerth J, Twaddle M (2011). Cultures of interdisciplinary teams: how to foster good dynamics. J Palliat Med.

[21] Tang CJ, Chan SW, Zhou WT, Liaw SY (2013). Collaboration between hospital physicians and nurses: an integrated literature review. Int Nurs Rev.

[22] Rawlinson C, Carron T, Cohidon C (2021). An overview of reviews on interprofessional collaboration in primary care: barriers and facilitators. Int J Integr Care.

[23] Salvage J, Smith R (2000). Doctors and nurses: doing it differently. BMJ.

[24] Sasaki H, Yonemoto N, Mori R, Nishida T, Kusuda S, Nakayama T (2016). Use of the ICU Nurse-Physician Questionnaire (ICU N-P-Q): testing reliability and validity in neonatal intensive care units in Japan. BMJ Open.

[25] Nancarrow SA, Booth A, Ariss S, Smith T, Enderby P, Roots A (2013). Ten principles of good interdisciplinary team work. Hum Resour Health.

[26] Morley L, Cashell A (2017). Collaboration in health care. J Med Imaging Radiat Sci.

[27] Penconek T, Tate K, Bernardes A (2021). Determinants of nurse manager job satisfaction: a systematic review. Int J Nurs Stud.

